# Pharmacokinetics, bioavailability and tissue distribution of chitobiose and chitotriose in rats

**DOI:** 10.1186/s40643-022-00500-y

**Published:** 2022-02-11

**Authors:** Mai Chen, Jiayang Jin, Xiaoguo Ji, Kunlin Chang, Juan Li, Liming Zhao

**Affiliations:** 1 School of Biotechnology, State Key Laboratory of Bioreactor Engineering, R&D Center of Separation and Extraction Technology in Fermentation Industry, East China University of Science and Technology, Shanghai, 200237 China; 2 Department of Nutrition, Chang-Zheng Hospital, Naval Medical University, Shanghai, 200003 China; 3 Shanghai Collaborative Innovation Center for Biomanufacturing Technology (SCICBT), Shanghai, 200237 China

**Keywords:** Chitobiose, Chitotriose, UPLC–MS, Pharmacokinetics, Tissue distribution

## Abstract

Chitooligosaccharides (COSs) have various physiological activities and broad application prospects; however, their pharmacokinetics and tissue distribution remain unclear. In this study, a sensitive and selective ultra-performance liquid chromatography–mass spectrometry (UPLC–MS) method for determining chitobiose (COS 2) and chitotriose (COS 3) in rat serum and tissues was developed. This method was successfully validated based on FDA guidelines in terms of selectivity, calibration curves (lower limit of quantification was 0.002 µg/mL for COS 2 and 0.02 µg/mL for COS 3), precision (intra-day relative standard deviation of 0.04%–3.55% and inter-day relative standard deviation of 1.94%–11.63%), accuracy (intra-day relative error of − 1.81%–11.06% and inter-day relative error of − 9.41%–8.63%), matrix effects, recovery (97.10%–101.29%), stability, dilution integrity, and carry-over effects. Then, the method was successfully applied to the pharmacokinetics and tissue distribution study of COS 2 and COS 3 after intragastric and intravenous administration. After intragastric administration, COS 2 and COS 3 were rapidly absorbed, reached peak concentrations in the serum after approximately 0.45 h, and showed rapid elimination with clearances greater than 18.82 L/h/kg and half-lives lower than 6 h. The absolute oral bioavailability of COS 2 and COS 3 was 0.32%–0.52%. COS 2 and COS 3 were widely distributed in Wistar rat tissues and could penetrated the blood–brain barrier without tissue accumulation.

**Graphic Abstract:**

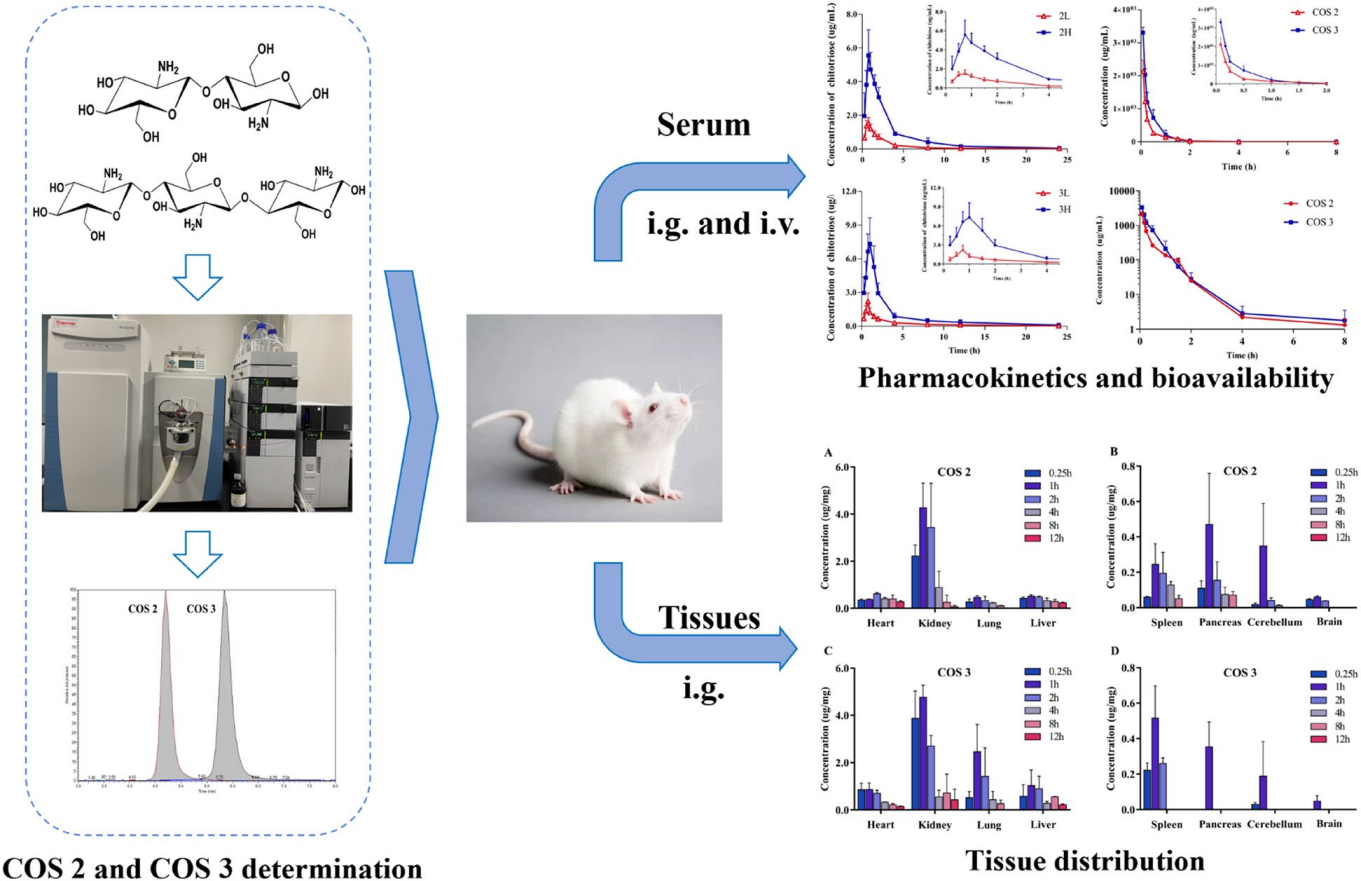

**Supplementary Information:**

The online version contains supplementary material available at 10.1186/s40643-022-00500-y.

## Highlights

- Report of tissue distribution of chitobiose and chitotriose after intragastric administration in rats.
- A trace detection method for the determination of chitobiose and chitotriose in rat serum and organs using UPLC–MS was developed and validated.
- Our results reveal that chitobiose and chitotriose could be quickly absorbed into the blood, with an oral bioavailability of 0.32%–0.52% in rats.

**Supplementary Information:**

The online version contains supplementary material available at 10.1186/s40643-022-00500-y.

## Introduction

Chitooligosaccharides (COSs) are oligomers produced chemically or enzymatically from chitosan, consisting of β-(1–4)-linked d-glucosamine and N-acetyl-d-glucosamine with a polymerization degree of 2–10 (Aam et al. 2010). COS have many excellent biochemical characteristics, including good solubility, biodegradability, biocompatibility, and low toxicity (Hao et al. 2021). COS also have antioxidant (Li et al. 2012; Mengibar et al. 2013), anti-inflammatory (Santos-Moriano et al. 2019), hypolipidemic (Deng et al. 2020; Lee et al. 2021), hypoglycemic (Kim et al. 2014), anti-Alzheimer’s disease (Ouyang et al. 2017), anti-tumor activity of colorectal cancer and orthotopic liver tumor (Mattaveewong et al. 2016; Zou et al. 2019), and other physiological effects (Huang et al. 2021; Lan et al. 2021). The relationship among COS, gut microbiota, and host health has received great attention, while in vivo and in vitro studies have shown that COS could reform the community structure of gut microbiota as a potential prebiotic (Ji et al. 2021a, b; Liu et al. 2020; Long et al. 2018). Such physiological activities make it suitable for many fields, such as nutraceutical and food additives as well as biomedical treatments. With its critical physiological benefits and broad applications, it is important to understand the bioavailability and dose–effect relationship of COSs.

Several studies have explored the intestinal absorption characteristics and tissue distribution of COS mixtures and chitosan (Chae et al. 2005; Chen et al. 2019; Zeng et al. 2008). Chae et al. showed that chitosan (3.8–230 kDa) can be absorbed in the epithelium of villi in the duodenum and jejunum compartment, and the absorption increases with a decrease in molecular weight (Chae et al. 2005). Another study showed that chitobiose (COS 2) and chitotriose (COS 3) could be absorbed into the blood, with a bioavailability of 0.7%–2.0% (Chen et al. 2005). COS 2 and COS 3 are not metabolized in the body, and more than 90% are eliminated from the urine after gavage (Chen et al. 2005). Further investigation of the absorption characteristics of COS in the Caco-2 cell line and everted gut sacs revealed that COS was absorbed by a combination of passive diffusion and active transport involving a sodium glucose cotransporter (SGLT) and glucosamine transporter (GLUT2) (Chen et al. 2019).

The detection of COS in biological samples is an essential part of pharmacokinetic studies, and the direct quantification of COS provides a more realistic picture of its disposition in vivo. Many analytical techniques have been used for COS quantification, including capillary electrophoresis (Hattori et al. 2010), fluorescence derivatization (Chen et al. 2005), high-performance anion-exchange chromatography with amperometric detection (Cao et al. 2016; Santos-Moriano et al. 2019), liquid chromatography-evaporative light scattering detection (LC-ELSD) (Chen et al. 2020), and liquid chromatography–mass spectrometry (LC–MS) (Regel et al. 2020). These methods are mainly focused on COS quantification in raw materials or enzymatic digests, but rarely in biological samples.

Fluorescent labeling has been widely used in COS detection to improve detection sensitivity. Zeng et al. (2008) studied the absorption and distribution of a COS mixture in mice after intragastric administration of fluorescein isothiocyanate (FITC)-labeled COS and showed that it was absorbed into the blood via the small intestine and rapidly distributed to the liver, kidney, thymus, spleen, lung, heart, and other tissues. In addition, in vitro and in vivo studies have shown that COS can cross the blood–brain barrier (Zhu et al. 2020a, b) and is widely distributed in vivo (Zhai et al. 2019) by detecting fluorescently labeled COS. However, this method greatly alters the molecular mass of COS. Many studies have shown that the physiological activity of COS is closely related to its molecular weight (He et al. 2020; Huang et al. 2021; Zhao et al. 2020; Zou et al. 2019); though whether fluorescent labeling alters the physiological activity and physical/chemical properties of COS has not been confirmed to date. Thus, the pharmacokinetics, bioavailability and tissue distribution of COS with a single degree of polymerization should be further investigated, and it is imperative to develop a sensitive and direct quantitation method for COS for pharmacokinetic analysis.

LC–MS is a powerful technique for the quantitative detection of compounds in biological samples because of its high sensitivity and specificity. Zhou et al. (2016) established a sensitive method for determining stachyose in rat plasma based on the LC–MS/MS method and successfully applied it to the pharmacokinetic study of stachyose. Jiang et al. (2020) quantitatively determined acarbose in plasma using the LC–MS technique, which was separated using a hydrophilic interaction chromatography column and applied the method to pharmacokinetic studies. However, a selective and sensitive method for determining COS in biological samples is yet to be developed to support pharmacokinetic studies and biodistribution.

The objective of the present study was to establish a rapid and sensitive UPLC–MS method for determining COS in bio-samples. We have selected two low-molecular weight chitooligosaccharides, COS 2 and COS 3, to elucidate the oral absolute bioavailability and pharmacokinetics of the two oligosaccharides at different doses and their tissue distribution characteristics.

## Materials and methods

### Chemicals and reagents

COS 2 (purity > 95%, Additional file 1: Figure S1a and c) and COS 3 (purity > 95%, Additional file 1: Figure S1b and d) were prepared in our laboratory, and their purities were measured and confirmed with HPLC-ELSD (Ji et al. 2021a, b). Acetonitrile and methanol were purchased from Fisher Scientific (Fair Lawn, NJ, USA). HPLC–MS-grade ammonia solution (25%, v/v) was purchased from Aladin (Shanghai, China). Ultrapure water was obtained from the Milli-Q system (Millipore, Bedford, MA, USA).

### Instrumentation and analytical conditions

The samples were analyzed on a LC-30A liquid chromatography system with a high-pressure LC-30AD pump, a SIL-30AC autosampler, and a CTO-30A column oven (Shimadzu Inc., Japan). The column eluates were analyzed using a Q-Exactive Plus mass spectrometer (MS) equipped with an Orbitrap mass analyzer with a heated electrospray ionization source (Thermo Fisher Scientific Inc., USA). The sequence and data analysis were performed using Xcalibur Qual Browser (Thermo Fisher Scientific Inc.).

The separation of the compound was performed using an ACQUITY UPLC Glycan BEH amide column (100 mm × 2.1 mm, 1.7 µm, Waters Corporation, MA, USA) combined with a Van Guard pre-column (5 mm × 2.1 mm, 1.7 µm, Waters Corporation) at 45 °C. The optimized method used a gradient mobile phase consisting of 0.1% (v/v) ammoniacal aqueous solution (solvent A) and 0.1% (v/v) ammonia in acetonitrile (solvent B). A flow rate of 0.2 mL/min was used with 2 µL of injection volume. The gradient conditions were 0.0–10.0 min, 75%–45% B; 10.0–10.1 min, 75% B; and 10.1–20 min 75% B to stabilize the initial conditions. The total run time was 10 min, and the post-delay time for reconditioning the column with 75% B was 10 min.

For the MS condition, the samples were detected in targeted-selected ion monitoring (t-SIM) for quantification with the m/z 341.1555 ([M + H]^+^) for COS 2 and m/z 502.2243 ([M + H]^+^) for COS 3, which operated in a positive ionization mode and the parameters settings were as described in the literature (Ardalani et al. 2021; Chitescu et al. 2015). High-purity nitrogen was used as both the ion source and collision gas. The quantitative parameters were as follows: resolution 70,000 FWHM (m/z 200) and automatic gain target 5 × 10^4^. The extracted ion chromatograms were used for quantitation, selecting m/z 341.1555 (mass tolerance < 6 × 10^–6^) and m/z 502.2243 (mass tolerance < 6 × 10^–6^) as the quantitative ions of COS 2 and COS 3, respectively.

### Preparation of standard solution and quality control sample

Stock solutions (1 mg/mL) of COS 2 and COS 3 for calibration and quality control were prepared in ultrapure water. A series of standard solutions were prepared, and equal amounts of COS 2 and COS 3 standard solutions were taken together to obtain a mixed standard solution.

To obtain the calibration curves, 5 µL samples of the mixed standard solution were mixed with 95 µL of blank serum or one of the tissue homogenate supernatants (liver, kidney, heart, lung, spleen, pancreas, cerebrum, and cerebellum). The COS 2 and COS 3 calibration curves consisted of seven non-zero concentrations in the range of 0.002–2.56 µg/mL and 0.020–5.12 µg/mL in serum and tissue homogenate supernatants. Quality control (QC) samples were independently prepared with blank serum at concentrations of 0.02 µg/mL (low), 0.20 µg/mL (medium), and 2.00 µg/mL (high) for COS 2 and 0.05 µg/mL (low), 0.50 µg/mL (medium), and 5.00 µg/mL (high) for COS 3. The high concentration serum samples were prepared by spiking COS 2 and COS 3 mixed standard solutions with blank serum to obtain concentrations of 2000 µg/mL and 200 µg/mL for COS 2, and 5000 µg/mL and 500 µg/mL for COS 3, which were 1000 times and 100 times higher than the high QC samples (2.00 µg/mL and 5.00 µg/mL for COS 2 and COS 3, respectively).

### Sample processing

The initial sample preparation was based on procedures previously described in the literature (Wang et al. 2020), and we simplified the protocol to maintain high throughput. Briefly, 100 µL of rat serum of pharmacokinetic samples (or QC samples) and 300 µL of methanol–acetonitrile (1:1, v/v) were vortexed for 30 s, and then centrifuged at 13,000× *g* for 10 min to precipitate the proteins. The supernatant was filtered through a 0.22 µm nylon syringe filter, and the liquid was transferred to a sample vial, and a 2-µL aliquot was injected for UPLC–MS analysis. Tissue samples were added to the cooled saline (1:5, w/v) and two grinding beads and subsequently homogenized in a homogenizer at 60 Hz for 120 s. After 30 s of vigorous vortexing, the sample was sonicated in an ice water bath for 10 min, centrifuged at 3000×*g* for 10 min at 4 °C, and 100 µL of supernatant was collected. The subsequent steps were the same as those for the serum.

### Method validation

The analytical method was validated according to U.S. Food and Drug Administration, Guidance for Industry–Bioanalytical Method Validation (Food and Drug Administration 2018).

Selectivity was assessed by comparing the chromatograms of six different batches of blank serum and tissue homogenization with the corresponding spiked serum and pharmacokinetic samples to ensure there were no interfering peaks (Jin et al. 2021).

A standard curve in the form of *y* = Ax + B was determined by plotting the peak areas of COS 2 and COS 3 against the known standard concentrations of COS 2 and COS 3. The slope, intercept, and coefficient of determination were estimated using the least squares linear regression method with a weighting of 1/x. The acceptance criterion was that the coefficient correlation (r^2^) must be greater than 0.990 (Zhou et al. 2021). The lower limit of quantification (LLOQ) is the lowest amount that can be quantitatively determined with a signal noise ratio 10:1.

The precision and accuracy of the method were evaluated by analyzing the COS 2 samples at concentrations of 0.02, 0.20, and 2.00 µg/mL and 0.05, 0.50, and 5.00 µg/mL for COS 3 samples. Five replicates on the same day were analyzed for the intra-day evaluation, and five replicates per day for two consecutive days were used for the inter-day evaluation to verify the repeatability of the method. The accuracy was obtained by comparing the measured values and theoretical values of the QC samples expressed as the relative error (RE). Acceptable levels of accuracy were 85%–115%. The relative standard deviation (RSD) was calculated to assess precision, which should be less than 15% (Zhu et al. 2020a, b).

The matrix effect and extraction recovery of COS 2 and COS 3 were determined at three levels in five replicates. The matrix effects were evaluated by comparing the peak area ratio of the post-extracted QC samples (spiked with analytes in the extracted analyte-free blank serum samples) to the peak area ratio of 75% acetonitrile solution. Recoveries were calculated by comparing the mean peak area of spiked QC samples with post-extracted QC samples (Ma and Wang 2021).

The stability of the analytes in rat serum was evaluated by investigating three QC concentrations in sextuplicate serum samples. The different storage and handling conditions were as follows: (a) autosampler tray stability at 10 °C for 24 h, and (b) freeze–thaw stability after three freeze–thaw cycles at − 20 °C (Penchala et al. 2013).

Dilution effects were used to determine the accuracy and precision of high concentration samples after dilution. Simulated high concentration serum samples were diluted 100-fold and 50-fold with post-dilution concentrations of 2 µg/mL for COS 2 and 5 µg/mL for COS 3. Blank serum was used for the dilutions and five replicates were performed. The RE and RSD values between the calculated and theoretical concentrations were measured to evaluate the accuracy and precision after dilution (Verougstraete et al. 2021).

Carry-over was performed in each analytical run by injecting three blank samples after the high QC sample (2 µg/mL for COS 2 and 5 µg/mL for COS 3). The outcomes of the carry-over effect should be less than or equal to 20% of the tested compounds (Ma and Wang 2021).

### Application to the pharmacokinetic study

The pharmacokinetic study was approved by the Institutional Animal Care and Use Committees of SHCQ (permit number SHCQ-20200032). Male Wistar rats (weight, 220 ± 10 g) were obtained from Shanghai SLAC Laboratory Animal Co., Ltd. (license NO. SCXK-hu-20170005). Prior to the start of the experiment, all rats were housed in animal rooms (temperature 22 ± 2 °C, humidity 40 ± 10%, 12 h light/dark cycle) with free access to water and food for a week acclimation period. A total of 24 rats were randomly divided into four groups and deprived of food with free access to water for 12 h before the experiments, and 100 mg/kg and 500 mg/kg of COS 2 and of COS 3 in pure water were intragastrically administered to rats. For intravenous experiments, 10 rats were randomly divided into two groups, and COS 2 and COS 3 were administered via the tail vein at a dose of 100 mg/kg separately to non-fasted rats. Blood samples (200 µL) were collected from the tail vein at 0.08, 0.17, 0.25, 0.5, 0.45, 1, 2, 4, 8, 12, and 24 h after intragastric dosing and 0.08, 0.17, 0.25, 0.5, 1, 1.5, 2, 4, and 8 h after intravenous dosing. Blood samples were left overnight at 4 °C and centrifuged at 3000×*g* at 4 °C for 10 min to obtain the serum.

### Tissue distribution study

A total of 60 rats were randomly divided into 12 groups (*n* = 5) and they were deprived of food with free access to water for 12 h prior to the experiments; six groups were intragastrically administered 500 mg/kg COS 2, and the remaining rats were treated with 500 mg/kg COS 3. Rats were killed at different times (0.25, 1, 2, 4, 8, and 12 h) by neck amputation and tissue samples (heart, liver, kidney, lung, spleen, pancreas, cerebellum, and brain) were collected. The tissue samples were rinsed with ice-cold 0.9% NaCl solution to remove blood or content and blotted on filter paper. Samples were snap-frozen in liquid nitrogen and stored at − 80 °C until subsequent processing.

### Statistical analysis

Each evaluation was repeated at least three times, and the results are expressed as mean values with standard deviation (SD). Charts were prepared using GraphPad Prism 7 (GraphPad Software Inc., San Diego, CA, USA). The pharmacokinetic parameters for each rat were estimated using Drug and Statistics software version 2.0. Non-compartmental analysis was used to determine the pharmacokinetic parameters including maximum serum concentration (*C*_max_), the area under the serum concentration–time curve (AUC), the half-life (*t*_1/2Z_), the mean residence time (MRT), clearance (Cl_Z/F_), and apparent volume of distribution (*V*_Z/F_) of COS 2 and COS 3.

## Results and discussion

### Method validation

#### Selectivity

**Fig. 1:**
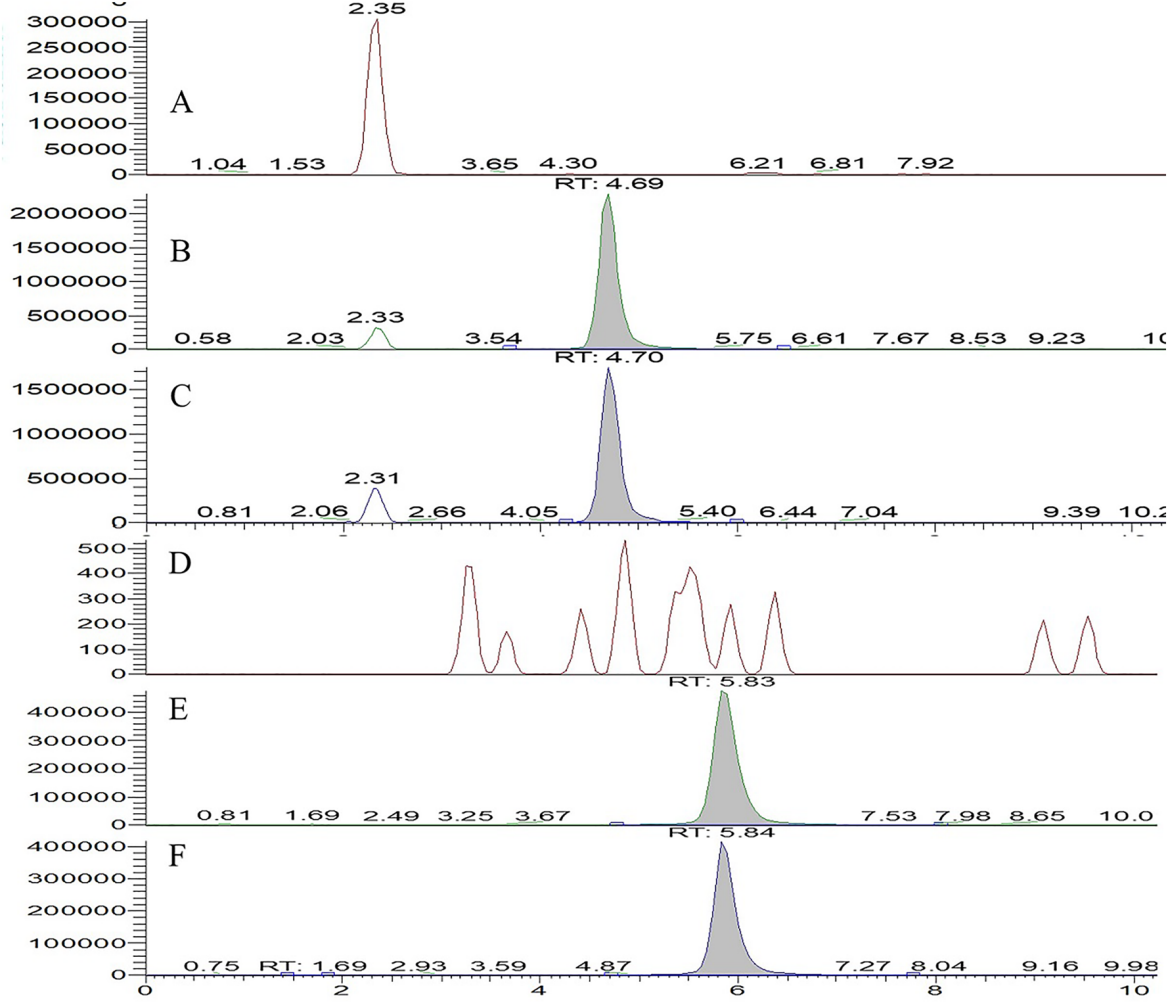
Typical SIM chromatograms for the determination of COS 2 and COS 3 in serum: blank matrix (**A**) (**D**); blank matrix spiked with COS 2 (**B**) and COS 3 (**E**); after intragastric administration of 500 mg/kg COS 2 (**C**) and after intragastric administration of 500 mg/kg COS 3 (**F**)

The typical selected ion monitoring (SIM) chromatograms of a blank serum sample, a blank serum sample spiked with COS 2 and COS 3, and pharmacokinetic samples after intragastric administration of 500 mg/kg COS 2 and COS 3 are shown in Fig. 1. No interfering endogenous substances were observed at the retention time of COS 2 and COS 3. In the SIM chromatograms of various tissues (heart, liver, kidney, lung, spleen, pancreas, cerebellum, and brain), no endogenous substances interfered with COS 2 and COS 3 detection (Additional file 1: Figures S2–S9), indicating that the selectivity assessment results met the FDA guidance of industry-bioanalytical method validation requirements (Food and Drug Administration 2018).

#### Linearity and lower limit of detection

The linearity of the calibration curves was found to be in the range of 0.002–2.56 µg/mL for COS 2 and 0.020–5.12 µg/mL for COS 3. The resulting standard curve equations for COS 2 and COS 3 in serum and various tissue homogenizations are shown in Table 1. The low limit of quantification was 0.002 µg/mL for COS 2 and 0.02 µg/mL for COS 3 with signal–noise ratio values greater than 10. The correlation coefficients (*r*^2^) of the standard curves were greater than 0.990, indicating that the linearity was good, and the quantitative analyses of COS 2 and COS 3 could be performed within their linear ranges.

**Table 1:** The standard curve, linear range, and r^2^ of COS 2 and COS 3 in serum and tissues

Analyte	Organs	Standard curve	r^2^	Linear range (μg/mL)
COS 2	Serum	y = 10000000x + 798,150	0.9992	0.002–2.560
	Heart	y = 20000000x-709953	0.9932	0.002–2.560
	Liver	y = 20000000x-573343	0.9962	0.002–2.560
	Kidney	y = 20000000x + 859,579	0.9971	0.002–2.560
	Lung	y = 10000000x-131031	0.9989	0.002–2.560
	Spleen	y = 10000000x-33949	0.9983	0.002–2.560
	Pancreas	y = 20000000x + 19,084	0.9985	0.002–2.560
	Cerebellum	y = 20000000x-19880	0.9934	0.002–2.560
	Brain	y = 20000000x-100188	0.9988	0.002–2.560
COS 3	Serum	y = 3000000x-200043	0.9984	0.020–5.120
	Heart	y = 2000000x-20788	0.9994	0.020–5.120
	Liver	y = 2000000x + 3060.5	0.9971	0.020–5.120
	Kidney	y = 3000000x + 60,188	0.9989	0.020–5.120
	Lung	y = 3000000x-88485	0.9928	0.020–5.120
	Spleen	y = 2000000x-53481	0.9979	0.020–5.120
	Pancreas	y = 3000000x + 140,458	0.9962	0.020–5.120
	Cerebellum	y = 3000000x + 38,595	0.9935	0.020–5.120
	Brain	y = 2000000x + 39,452	0.9974	0.020–5.120

#### Matrix effect and recovery

As shown in Table 2, the means of extraction recoveries of COS 2 and COS 3 for QC samples at low, medium, and high concentrations were between 97.10 and 101.29% with RSD values within 10.79%, and there was no significant difference between the extraction recoveries of COS 2 and COS 3, indicating that the method had good extraction recoveries. The mean values of the matrix effects for COS 2 and COS 3 at the three concentrations ranged from 89.79 to 94.95% with RSD values within 7.83%, indicating that the ion-enhancing or ion-inhibiting effects of serum endogenous substances were negligible.

**Table 2:** Extraction recovery and matrix effect of COS 2 and COS 3 in serum

Analyte	Nominal concentration (μg/mL)	Recovery (%)		Matrix effect (%)	
		(Mean ± SD, *n* = 5)	RSD	(Mean ± SD, *n* = 5)	RSD
COS 2	2.00	101.29 ± 7.64	7.54	89.79 ± 1.52	1.69
	0.20	97.91 ± 3.16	3.23	92.92 ± 3.44	3.70
	0.02	99.28 ± 2.43	2.45	94.95 ± 1.41	1.48
COS 3	5.00	97.10 ± 7.39	7.61	90.41 ± 4.41	4.88
	0.50	97.52 ± 5.36	5.49	89.23 ± 4.11	4.60
	0.05	97.85 ± 10.56	10.79	92.95 ± 7.28	7.83

#### Accuracy and precision

The results obtained from the analysis of the accuracy and precision of QC samples at the three concentration levels are summarized in Table 3. The intra-day and 2-day inter-day accuracy (RE%) for COS 2 varied from − 9.41% to 7.38%, and the precision (RSD%) varied from 0.04% to 7.09%. For COS 3, the RE values of intra-day and 2-day inter-day precision were between − 2.38 and 11.06%, whereas the RSD values of accuracy ranged from 0.05 to 11.63%. These results were within the limits of accuracy and precision acceptable for the corresponding criteria (Food and Drug Administration 2018), indicating that the method was accurate and precise for the quantitative analysis of COS 2 and COS 3.

**Table 3:** Intra-day and inter-day accuracy and precision of COS 2 and COS 3 in serum

Analyte	Nominal concentration (μg /mL)	Intra-day (*n* = 5)			Inter-day (*n* = 5)			Inter-day (*n* = 5)			
		Calculated concentration (µg /mL)	Accuracy (RE%)	Precision (RSD%)	Calculated concentration (µg /mL)	Accuracy (RE%)	Precision (RSD%)	Nominal concentration (µg /mL)	Calculated concentration (µg /mL)	Accuracy (RE%)	Precision (RSD%)
COS 2	2.	2. ± 0.073	1.39	.55	1.81 ± 0.04	− 9.41	1.94	2.00009503	1. ± 0.12	− 2.36	6.Accuracy (RE%)05
	0.20	0.20 ± 0.01	− 1.87	0.04	0.20 ± 0.01	0.16	5.62	0.20	0.20 ± 0.01	0.23	4.03
	0.02	0.02 ± 0.00	2.59	0.08	0.02 ± 0.00	7.38	7.09	0.02	0.02 ± 0.00	− 2.12	4.08
COS 3	5.00	5.19 ± 0.36	3.84	0.07	5.08 ± 0.59	1.60	11.63	5.00	4.88 ± 0.20	− 2.38	4.00
	0.50	0.55 ± 0.03	9.10	0.05	0.53 ± 0.01	6.52	2.60	0.50	0.49 ± 0.05	− 2.03	9.20
	0.05	0.06 ± 0.00	11.06	0.07	0.05 ± 0.00	8.63	7.38	0.05	0.05 ± 0.00	5.35	3.43

#### Stability

The precision and accuracy of the serum samples after 24 h and three freeze–thaw cycles in the autosampler at 10 °C are shown in Table 4. The accuracy values of COS 2 and COS 3 at the three concentrations ranged from − 6.93 to 14.30%, and their precision values were less than 10.76%, which met the corresponding requirements (Food and Drug Administration 2018). Therefore, samples containing COS 2 or COS 3 at different concentrations had short- and long-term stability.

**Table 4:** Stability results of COS 2 and COS 3 under two different storage conditions

Stability test	Analyte	Nominal concentration (μg/mL)	Calculated concentration (μg/mL)	Accuracy (RE%)	Precision (RSD%)
Three freeze–thaw cycles	COS 2	2.00	1.88 ± 0.15	− 5.81	7.89
		0.20	0.19 ± 0.01	− 3.49	3.71
		0.02	0.02 ± 0.00	− 1.97	7.41
	COS 3	5.00	4.87 ± 0.35	− 2.69	7.23
		0.50	0.49 ± 0.05	− 2.40	9.28
		0.05	0.06 ± 0.00	14.16	6.61
Autosampler for 24 h	COS 2	2.00	1.86 ± 0.05	− 6.93	2.50
		0.20	0.20 ± 0.00	1.11	2.20
		0.02	0.02 ± 0.00	6.80	7.09
	COS 3	5.00	5.20 ± 0.56	4.03	10.76
		0.50	0.51 ± 0.04	1.15	8.36
		0.05	0.06 ± 0.00	14.30	3.81

#### Dilution effects

The simulated high concentration COS 2 and COS 3 serum samples were diluted 1000-fold and 100-fold by blank serum to assess dilution reliability, with the results shown in Table 5. The accuracy of COS 2 and COS 3 after dilution was in the range of − 4.71% to 5.16%, and the precision was less than 4.97% for both. Therefore, the determination of the simulated high concretion COS 2 and COS 3 serum samples after diluted 1000 times and 100 times by blank serum did not affect the precision and accuracy of the analytical method; and the diluted results were reliable.

**Table 5:** The dilution effects of COS 2 and COS 3 in serum

Analyte	Spiked concentration (μg/mL)	Dilution factor	Calculated concentration (μg/mL)	Accuracy (RE%)	Precision (RSD%)
COS 2	2000.00	1000	2.10 ± 0.01	5.16	0.62
	200.00	100	2.05 ± 0.02	2.70	1.13
COS 3	5000.00	1000	4.76 ± 0.24	− 4.71	4.97
	500.00	100	4.80 ± 0.23	− 4.06	4.84

#### Carry-over effect

No corresponding peak was detected near the retention time of the analyte in the blank serum sample that was analyzed after the high QC sample. The results showed that no carry-over effect was observed in this method, as shown in Additional file 1: Figure S10.

### Pharmacokinetic study

**Fig. 2:**
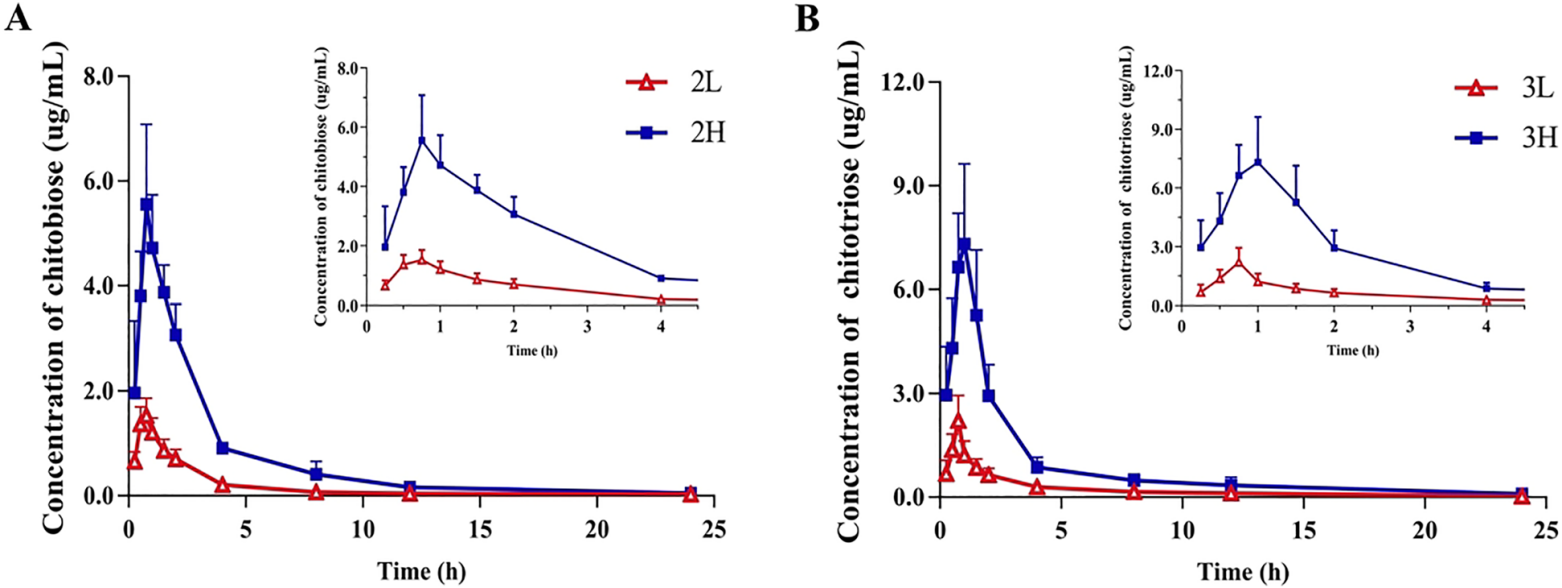
Pharmacokinetic profile of COS 2 (**A**) and COS 3 (**B**) concentration (μg /mL) in rat serum (*n* = 6) vs a withdrawal time after 100 and 500 mg/kg intragastric administration (2L: 100 mg/kg of COS 2; 2H: 500 mg/kg of COS 2; 3L: 100 mg/kg of COS 3; 3H: 500 mg/kg of COS 3; *n* = 6)

The validated method was successfully applied to investigate the serum concentration profiles of COS 2 and COS 3 in rats after intravenous and intragastric administration of COS 2 and COS 3. The average concentration–time profiles after intragastric administration are shown in Fig. 2. The pharmacokinetic parameters were calculated using non-compartmental analysis in DAS 2.0 software and the main parameters are summarized in Table 6. After intragastric administration of different doses of COS 2, it was rapidly absorbed into the blood and reached its peak concentration within 1 h, similar to COS 3. The peak time of glucosamine in serum after oral administration was about 0.8 h (Ibrahim et al. 2012), which was consistent with COS 2 and COS 3, indicating they can be quickly absorbed into the blood after oral administration. The AUC_0-t_ and *C*_max_ of COS 2 and COS 3 at high doses were higher than those at low doses (as shown in Table 6), indicating that their absorption process in rats was dose-dependent. *C*_max_/Dose and AUC_0-t_/Dose were calculated to compare the ability of COS 2 and COS 3 to be absorbed at the same dose. The calculated C_max_/Dose ranking was 3L > 3H > 2L > 2H, and the ranking of AUC_0-t_/Dose values was 3L > 2L > 3H > 2H, indicating better absorption of COS 3 than COS 2 and better absorption at lower doses than higher doses. It could be observed from Fig. 2 that there is no double-peak phenomenon displayed by the analytes, and we speculated that the analytes did not have enterohepatic circulation. A significantly higher apparent volume of distribution (V_Z/F_ values greater than 134.33 L/kg for both COS 2 and COS 3) than the total body water of rat (0.69 L/kg) (Davies and Morris 1993) suggests that COS 2 and COS 3 had a high distribution into the body tissues. The half-life of COS 2 and COS 3 were in the range of 3.95 h to 5.90 h, which were longer than the half-life of glucosamine in the range of 1.04 h to 2.84 h (Ibrahim et al. 2012). And the MRT_0-t_ of COS 2 and COS 3 were within 6 h and the Cl _Z/F_ values were greater than 18.82 L/h/kg at both low and high doses; therefore, the plasma concentrations of COS 2 and COS 3 decreased quickly.

**Table 6:** Pharmacokinetic parameters of COS 2 and COS 3 after intragastric administration and intravenous administration (data derived from non-compartmental model, mean ± SD, *n* = 6)

Parameters	Unit	i.g. COS 2		i.g. COS 3		i.v. COS 2	i.v. COS 3
		100 mg/kg	500 mg/kg	100 mg/kg	500 mg/kg	100 mg/kg	100 mg/kg
AUC_0-t_	mg/L·h	4.09 ± 0.68	16.38 ± 2.19	4.81 ± 0.65	20.01 ± 4.29	820.24 ± 67.60	1340.53 ± 130.92
AUC_0-∞_	mg/L·h	4.30 ± 1.08	16.61 ± 2.32	5.40 ± 0.76	21.44 ± 5.51	821.81 ± 68.03	1340.55 ± 130.93
MRT_0-t_	h	4.12 ± 1.21	3.71 ± 0.82	4.14 ± 1.60	4.02 ± 1.47	0.44 ± 0.06	0.38 ± 0.11
MRT_0-∞_	h	6.49 ± 3.95	4.20 ± 0.94	6.57 ± 2.45	5.63 ± 3.00	0.46 ± 0.07	0.39 ± 0.11
t_1/2z_	h	5.52 ± 6.50	3.95 ± 1.84	5.09 ± 1.83	5.90 ± 3.52	0.84 ± 0.33	0.58 ± 0.12
T_max_	h	0.75 ± 0.00	0.79 ± 0.10	0.71 ± 0.10	0.92 ± 0.13	0.08 ± 0.00	0.08 ± 0.00
CL_Z/F_	L/h/kg	24.43 ± 5.81	30.63 ± 4.58	18.82 ± 2.64	24.84 ± 7.41	0.12 ± 0.01	0.08 ± 0.01
V_Z/F_	L/kg	162.32 ± 136.07	167.10 ± 64.50	134.33 ± 38.50	189.30 ± 78.96	0.15 ± 0.06	0.06 ± 0.02
C_max_	mg/L	1.53 ± 0.34	5.61 ± 1.46	2.33 ± 0.60	7.88 ± 1.87	2137.85 ± 335.25	3312.24 ± 160.94
F_0-∞_	%	0.52	0.40	0.40	0.32	N/A	N/A

i.g., intragastric administration; i.v., intravenous administration

**Fig. 3:**
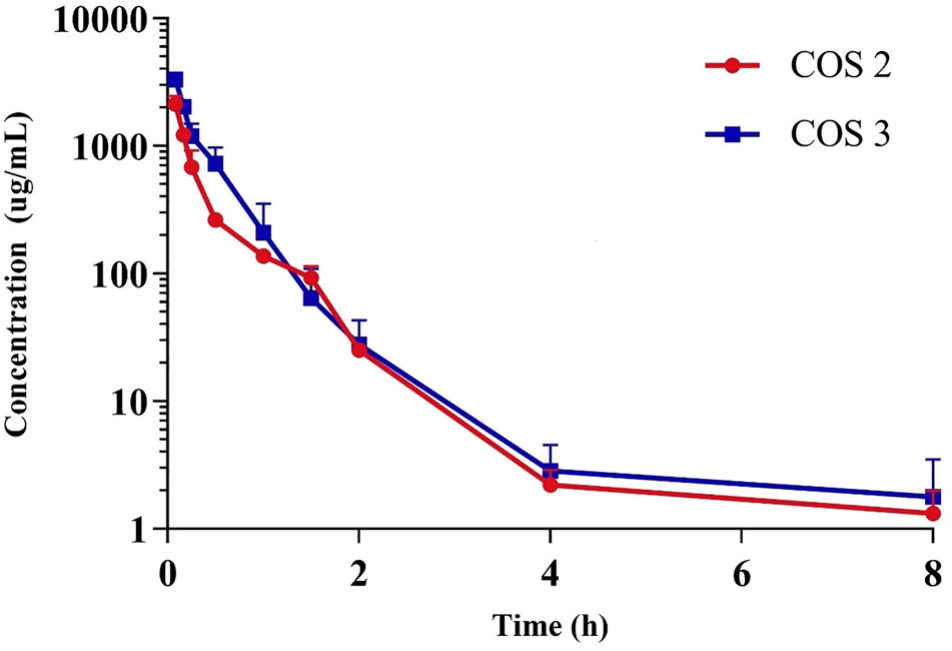
Mean serum concentration–time profile of COS 2 and COS 3 in rat serum after 100 mg/kg intravenous administration (*n* = 5)

The mean plasma concentration–time curves of COS 2 and COS 3 after intravenous administration at a dose of 100 mg/kg are shown in Fig. 3. Concentrations of COS 2 and COS 3 were measurable for up to 8 h after injection and its concentration range in serum was comparable to that reported in a previous study (Chen et al. 2005). After intravenous injection of COS 2 and COS 3, *t*_1/2_ was less than 1 h and MRT_0-t_ was less than 0.5 h, indicating that they were quickly cleared in vivo. The oral absolute bioavailability of COS 2 and COS 3 was calculated as 0.32%–0.52% using the equation *F* = (AUC i.g. × Dose i.v.)/(AUC i.v. × Dose i.g.) × 100%. Compartment model was used to analyze intravenous injection data and the lowest Akaike information criterion (AIC) data were used as compartment model analysis standard. The DAS software was used to calculate the AIC under different compartment models and different weighting factors. And results show that COS 2 and COS 3 have the lowest AIC value in the 1/Y^2^ weighting factor of the two-compartment model (Table 7). The pharmacokinetic parameters calculated by the selected compartment model are shown in Table 8. Chen et al. stated that COS 2 and COS 3 were one-compartment model through intravenous injection data with a sampling time of 0–2 h which may miss the distribution period (Zhao et al. 2020). To improve on this, the sampling time of this study was extended to 8 h, and results showed that COS 2 and COS 3 met the two-compartment model characteristics.

**Table 7:** Akaike information criterion (AIC) values of compartment model of rats with intravenous administration of COS 2 and COS 3

Analyte	Number of compartment	Weight factor	AIC value (mean ± SD)	Number of compartment	Weight factor	AIC value (mean ± SD)
COS 2	1	1	101.08 ± 4.41	2	1	92.36 ± 10.97
	1	1/C	54.62 ± 3.41	2	1/C	41.43 ± 4.58
	1	1/C^2^	11.23 ± 1.01	2	1/C^2^	9.94 ± 0.45
COS 3	1	1	114.05 ± 5.86	2	1	103.29 ± 11.83
	1	1/C	55.68 ± 5.39	2	1/C	45.37 ± 9.95
	1	1/C^2^	12.05 ± 3.16	2	1/C^2^	3.07 ± 6.44

**Table 8:** Main pharmacokinetic parameters of COS 2 and COS 3 after intravenous administration (mean ± SD, *n* = 5, data from compartment model)

Parameters	Unit	Values (mean ± SD)	
		COS 2	COS 3
t_1/2α_	h	0.08 ± 0.04	0.16 ± 0.08
t_1/2β_	h	0.52 ± 0.04	1.48 ± 0.97
V_1_	L/kg	0.02 ± 0.01	0.03 ± 0.01
CL	L/h/kg	0.09 ± 0.01	0.06 ± 0.00
AUC_0-t_	mg/L·h	889.00 ± 84.43	1386.34 ± 105.09
AUC_0-∞_	mg/L·h	1148.84 ± 141.98	1764.47 ± 93.07
K10	1/h	4.64 ± 2.24	2.42 ± 1.16
K12	1/h	5.98 ± 3.36	3.22 ± 4.55
K21	1/h	2.13 ± 0.32	1.15 ± 1.61

Chen et al. (2020) evaluated the possibility that digestion process would change the structure of COSs through in vitro static and dynamic digestion models. It was indicated that COS with DP 2–5 was resisted to low pH and digestive enzyme during gastric and intestinal digestion (Zhao et al. 2020). After COS 2 and COS 3 reached the intestine, they could be absorbed by the epithelium in the duodenum, jejunum and ileum (Chae et al. 2005; Chen et al. 2019). Studies have shown that the apparent permeability coefficient (Papp) of COS calculated in everted gut sacs was greater than 1 × 10^–6^ cm/s (Chen et al. 2019) and was completely absorbed (Liu et al. 2017). The COS concentration used to determine Papp in everted gut sacs was 2.00 mg/mL, which was much lower than the concentrations used in the present study (46.00 mg/mL and 230.00 mg/mL). Passive diffusion and active transport dominated the absorptive process of COS, with active transport involving GLUT2 and SGLT. Also, GLUT2 and SGLT are the main transporter proteins involved in the intestinal absorption of oligosaccharides such as glucose and glucosamine. To avoid the interference of COS absorption, we fasted for 12 h before the gavage and intravenous injection experiments. The involvement of transporters in COS absorption raises the possibility of the existence of a capacity-limited intestinal absorption of the compound, which may induce low bioavailability.

Due to the high polarity and poor lipophilicity of COS, their bioavailability was low in vivo (Naveed et al. 2019). Roman et.al (2019) used FAF-Drugs, SwissADME, and admesSAR 2.0 tools to predict the pharmacokinetic profiles of COS and results showed the oral bioavailability decreases with increasing molecular weight. Our results confirmed this prediction that the COS 3 being less bioavailable than COS 2, and both were lower than the oral bioavailability of glucosamine (6%) (Ibrahim et al. 2012), indicating limited absorption when administered in a monomeric form. With low absorption into blood by intestine, COS 2 and COS 3 can act as prebiotics for beneficial bacteria (Ji et al. 2021a, b; Liu et al. 2020).

### Tissue distribution

**Fig. 4:**
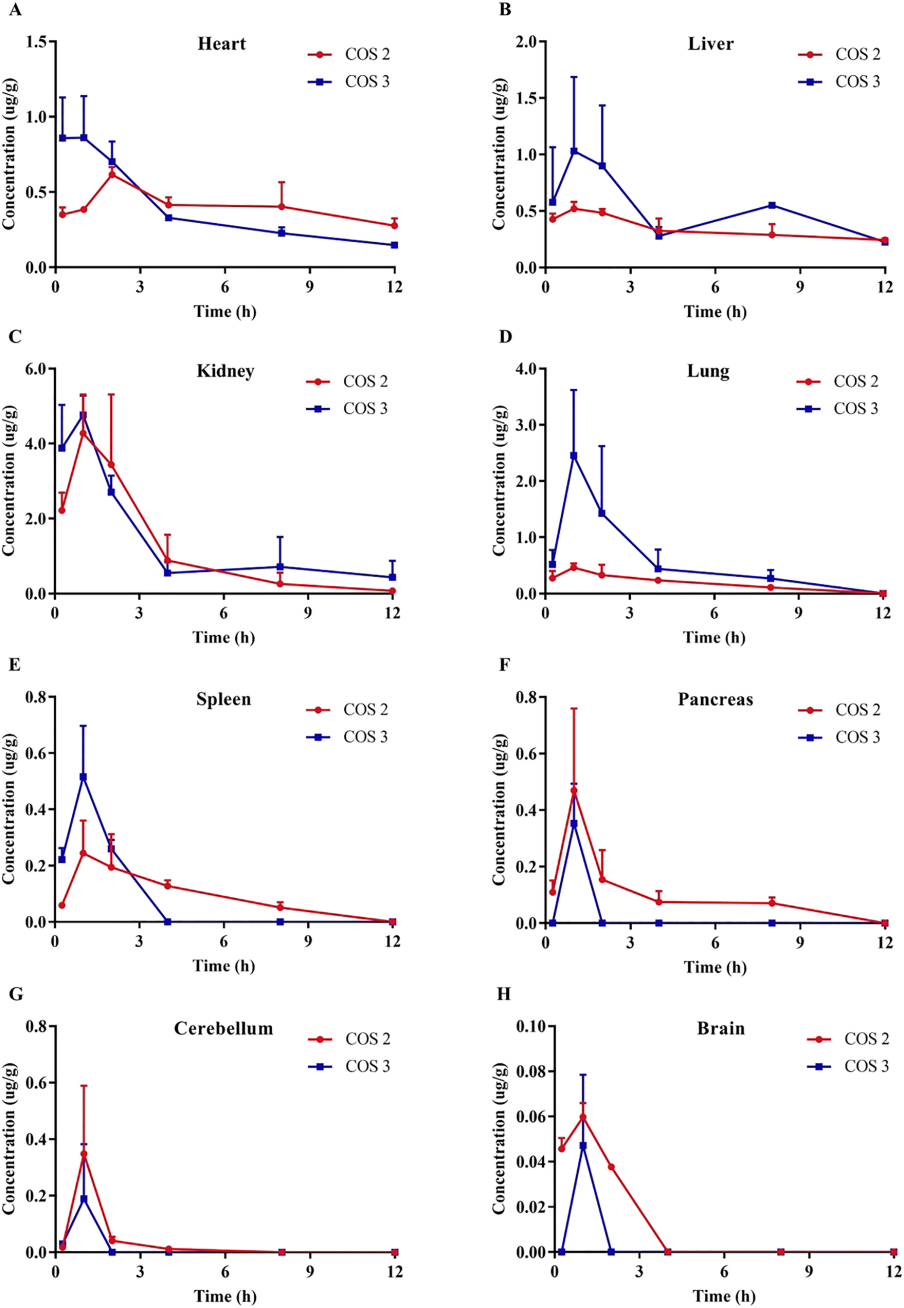
Distribution of COS 2 and COS 3 in various tissues after intragastric administration (**A**-heart, **B**-liver, **C**-kidney, **D**-lung, **E**-spleen, **F**-pancreas, **G-**cerebellum, **H**-brain; *n* = 5)

The mean tissue concentration–time profiles of COS 2 and COS 3 after intragastric administration are shown in Fig. 4, and the mean pharmacokinetic parameters are listed in Table 9. COS 2 and COS 3 were detected in most tissues at 0.25 h after intragastric administration and reached a peak concentration in the tissues after approximately 1 h. COS 2 and COS 3 were widely distributed in the heart, liver, kidney, lungs, spleen, pancreas, cerebellum, and brain, explaining the large apparent distribution volume estimated from the plasma concentration data. As shown in Table 9, COS 2 and COS 3 had the highest AUC_0-t_ and C_max_ values in the kidney; as found in previous studies, they were mainly eliminated through the kidney (Cao et al. 2016; Chen et al. 2005), probably due to their good water solubility. Neither COS 2 nor COS 3 were found in high concentrations in the liver, possibly indicating that neither had a significant first-pass effect in the liver.

**Table 9:** Main pharmacokinetic parameters of COS 2 and COS 3 in rat tissues after intragastric administration (*n* = 5)

Analyte	Organs	C_max_ (mg/L)	T_max_ (h)	AUC_0-t_ (mg/L·h)	AUC_0-∞_ (mg/L·h)	MRT_0-t_ (h)	t_1/2z_ (h)
COS 2	Heart	0.62 ± 0.05	2.00 ± 0.00	4.84 ± 0.76	12.46 ± 4.33	5.50 ± 0.27	13.43 ± 4.55
	Liver	0.53 ± 0.05	1.33 ± 0.58	4.02 ± 0.32	7.08 ± 2.01	5.21 ± 0.40	9.18 ± 3.58
	Kidney	4.33 ± 1.13	1.33 ± 0.58	13.82 ± 4.68	14.02 ± 4.53	2.48 ± 0.21	1.75 ± 0.55
	Lung	0.52 ± 0.03	1.33 ± 0.58	1.96 ± 0.26	2.51 ± 0.37	3.08 ± 0.19	3.52 ± 0.68
	Spleen	0.31 ± 0.08	1.33 ± 0.58	1.02 ± 0.16	1.35 ± 0.33	3.11 ± 0.27	3.88 ± 2.09
	Pancreas	0.47 ± 0.29	1.00 ± 0.00	1.07 ± 0.13	1.84 ± 0.90	2.80 ± 0.80	4.37 ± 3.26
COS 3	Heart	1.04 ± 0.12	0.5 ± 0.43	4.43 ± 0.41	5.40 ± 0.86	4.01 ± 0.14	5.18 ± 1.75
	Liver	1.41 ± 0.26	1.08 ± 0.88	6.04 ± 1.18	8.54 ± 1.17	5.03 ± 0.33	8.26 ± 7.48
	Kidney	4.85 ± 0.58	0.75 ± 0.43	15.53 ± 3.75	17.96 ± 7.70	3.16 ± 1.40	2.88 ± 1.95
	Lung	2.99 ± 0.25	1.33 ± 0.58	6.40 ± 0.48	7.06 ± 1.16	2.49 ± 0.44	1.85 ± 0.84

Some tissues did not have at least 5 concentrations for non-compartmental model analysis calculated by DAS software, thus having no pharmacokinetic parameters

From the data in Table 9, the *C*_max_ and AUC of COS 3 in the lungs were 5.75 and 3.26 times higher than COS 2, respectively. COS 2 was still detected in the pancreas at 8 h after administration; however, COS 3 was only detected at 1 h after administration. Differences in tissue distribution might lead to differences in their physiological activities. Hattori et al. (2010) qualitatively showed that COS can penetrate the blood–brain barrier as proved by fluorescence labeling. In this study, COS 2 and COS 3 were directly measured in rat serum and various tissues by the UPLC–MS method which we established, confirming that they can penetrate the blood–brain barrier (as shown in Fig. 4). However, the concentration was very low and cleared rapidly within 4 h, indicating that COS 2 and COS 3 had low blood–brain barrier penetration. COS 2 and COS 3 concentrations were low in all tissues, and the MRT_0-t_ was less than 6 h, indicating no accumulation effect.

## Conclusions

In conclusion, in order to detect the pharmacokinetics, bioavailability and tissue distribution of COS 2 and COS 3, a sensitive and accurate UPLC–MS method was first successfully developed and validated to determine COS 2 and COS 3 levels in rat serum and tissues. The pharmacokinetic results indicated that COS 2 and COS 3 could be rapidly absorbed into the blood at a peak concentration within 1 h. However, the values of compounds that could be absorbed into the blood by intragastric administration was limited to the bioavailability in the range of 0.32%–0.52%. After gavage, COS 2 and COS 3 were quickly distributed in the heart, liver, kidney, lung, spleen, and pancreas and penetrated the blood–brain barrier without tissue accumulation. The present study provides useful information that can further advance physiological research on COS and expand its application.

### Supplementary Information

**Additional file 1.** Additional figures.

## Data Availability

All data generated or analyzed during this study are included in this published article and its additional information files.

## Abbreviations

UPLC–MS: Ultra-performance liquid chromatography–mass spectrometry
COS: Chitooligosaccharides
COS 2: Chitobiose
COS 3: Chitotriose
FDA: Food and Drug Administration
SGLT: Sodium glucose cotransporter
GLUT2: Glucosamine transporter
LC-ELSD: Liquid chromatography-evaporative light scattering detection
LC–MS: Liquid chromatography–mass spectrometry
FITC: Fluorescein isothiocyanate
QC: Quality control
RE: Relative error
RSD: Relative standard deviation
AUC: Area under the serum concentration–time curve
t_1/2Z_: Half-life
MRT: Mean residence time
Cl_Z/F_: Clearance
V_Z/F_: Apparent volume of distribution
AIC: Akaike information criterion
