# Supplementary material for: Pharmacokinetics, bioavailability and tissue distribution of chitobiose and chitotriose in rats
Source: Bioresour Bioprocess. 2022 Feb 11;9(1):13. doi: 10.1186/s40643-022-00500-y (PMC10991139; doi:10.1186/s40643-022-00500-y)
Supplement: Supplementary file 1 — Additional file 1. Additional figures. [file 40643_2022_500_MOESM1_ESM.docx]

**Supplementary figures**


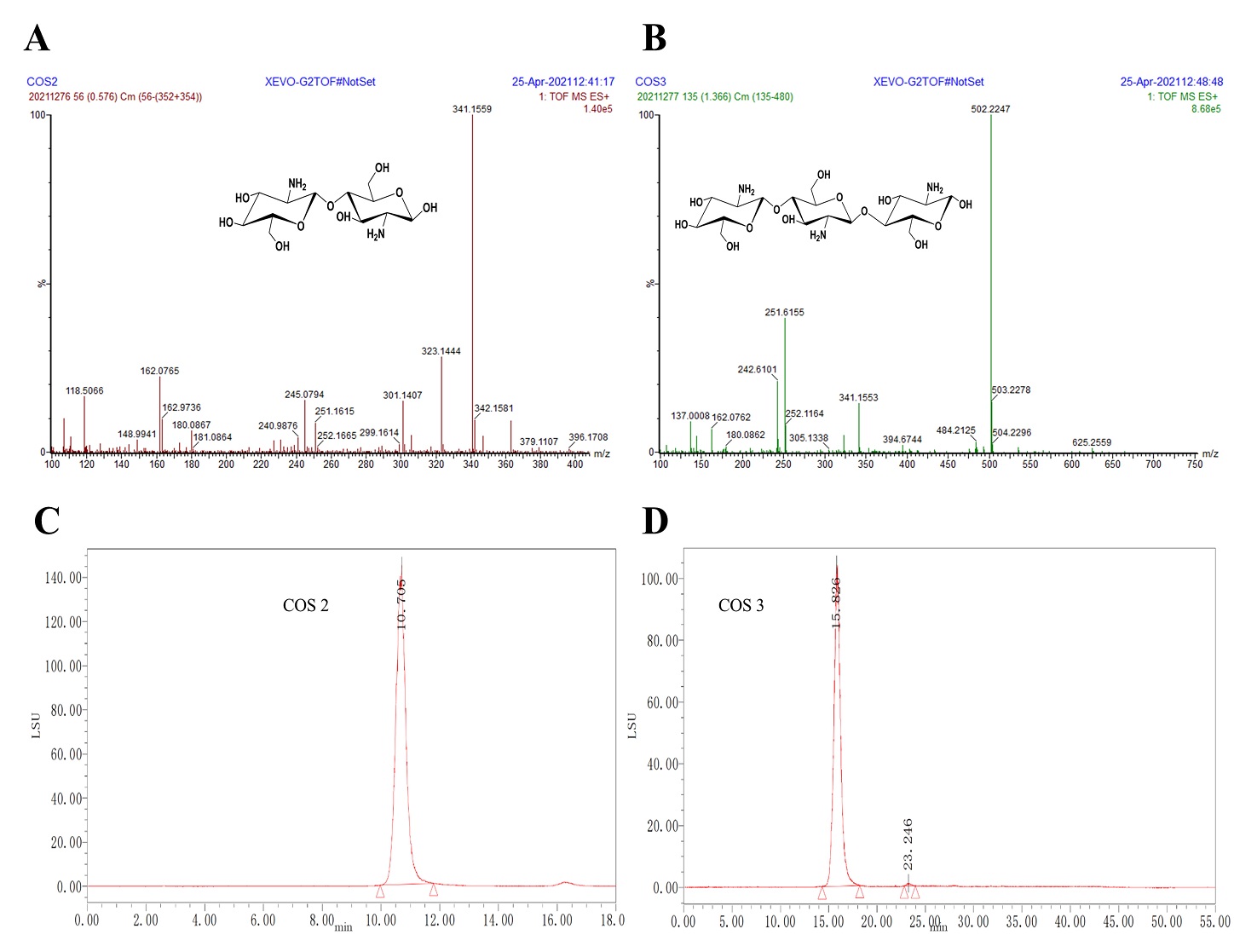


**Figure S1. Mass spectrum of chitobiose (A); mass spectrum of chitotriose (B); HPLC chromatograms of COS 2 (5 mg/mL) (C); HPLC chromatograms of COS 3 (5 mg/mL) (D).**

**
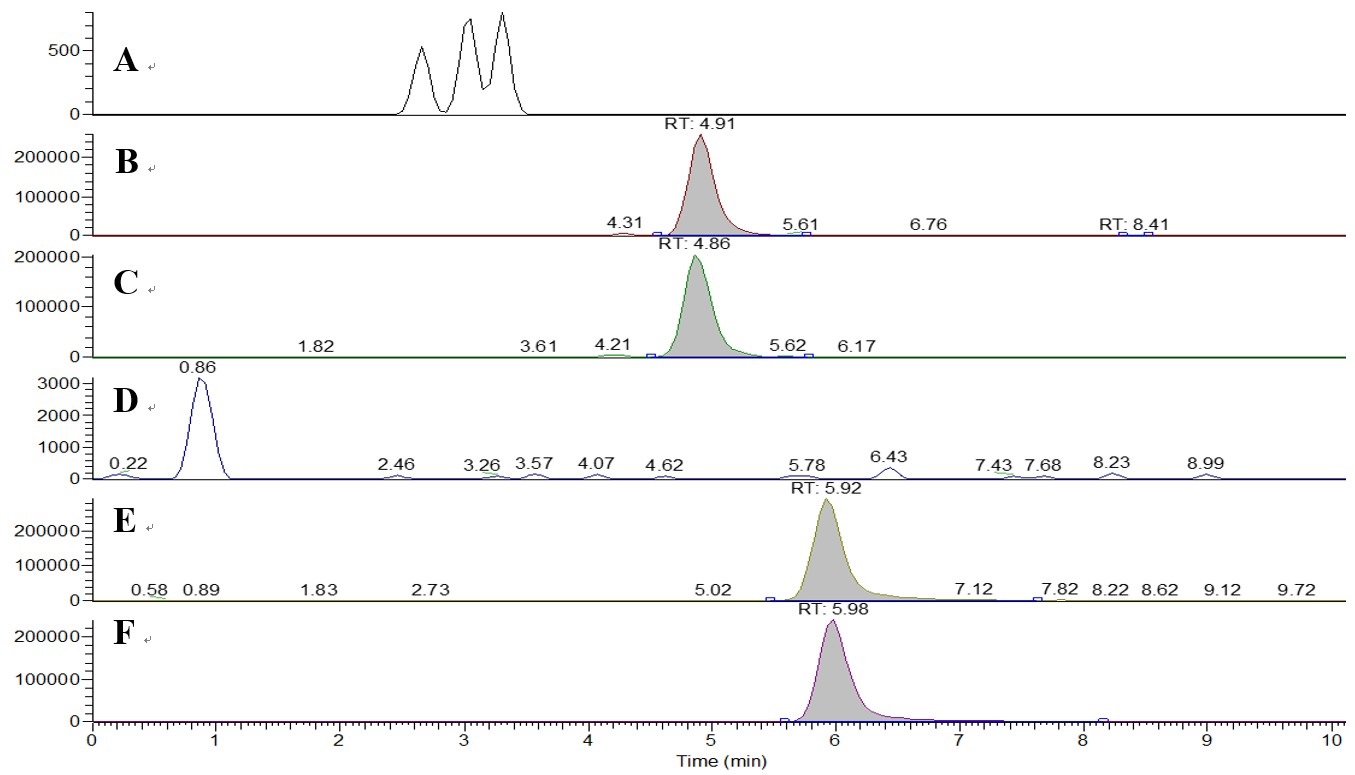
**

**Figure S2. Typical SIM chromatograms for the determination of COS 2 and COS 3 in heart: blank matrix (A) (D); blank matrix spiked with COS 2 (B) and COS 3 (E); after intragastric administration of 500 mg/kg COS 2 (C) and after intragastric administration of 500 mg/kg COS 3 (F).**

**
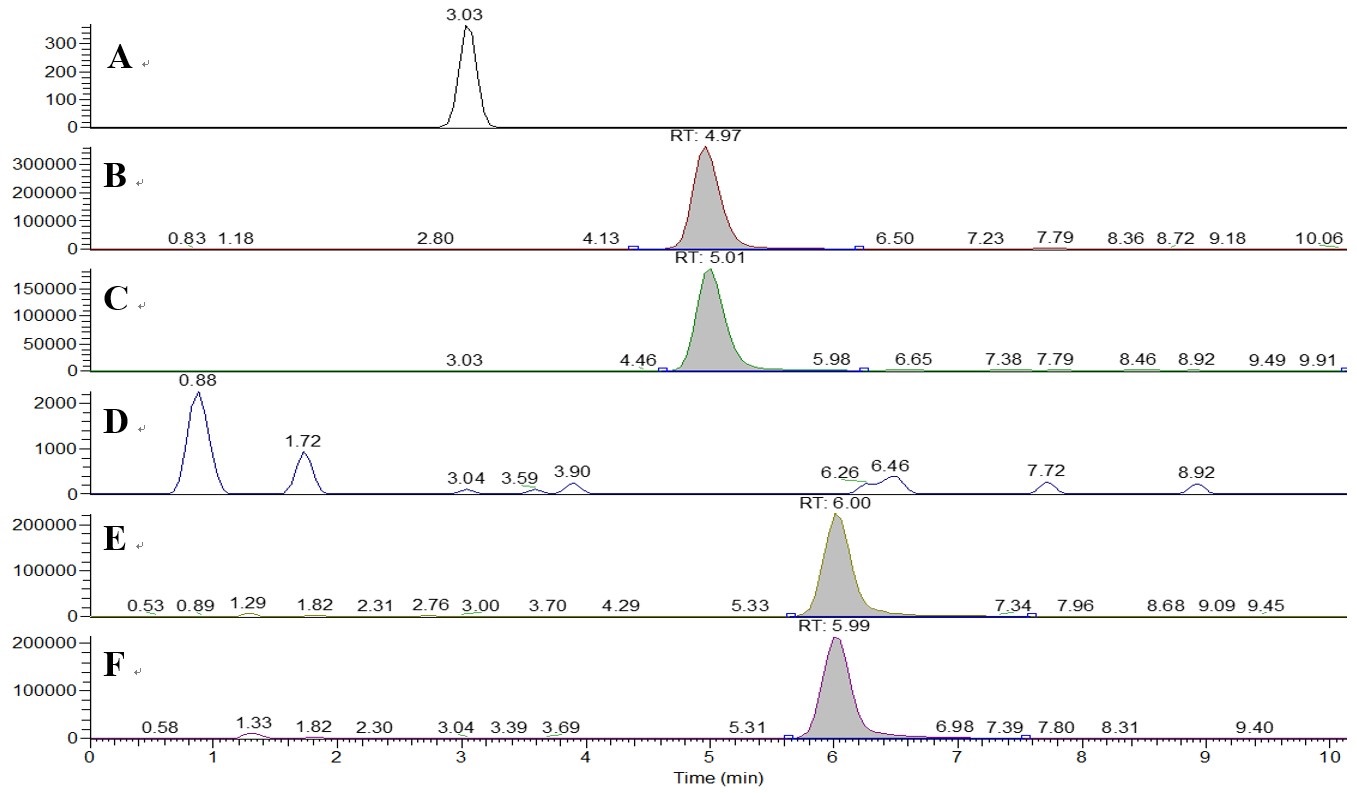
**

**Figure S3. Typical SIM chromatograms for the determination of COS 2 and COS 3 in liver: blank matrix (A) (D); blank matrix spiked with COS 2 (B) and COS 3 (E); after intragastric administration of 500 mg/kg COS 2 (C) and after intragastric administration of 500 mg/kg COS 3 (F).**

**
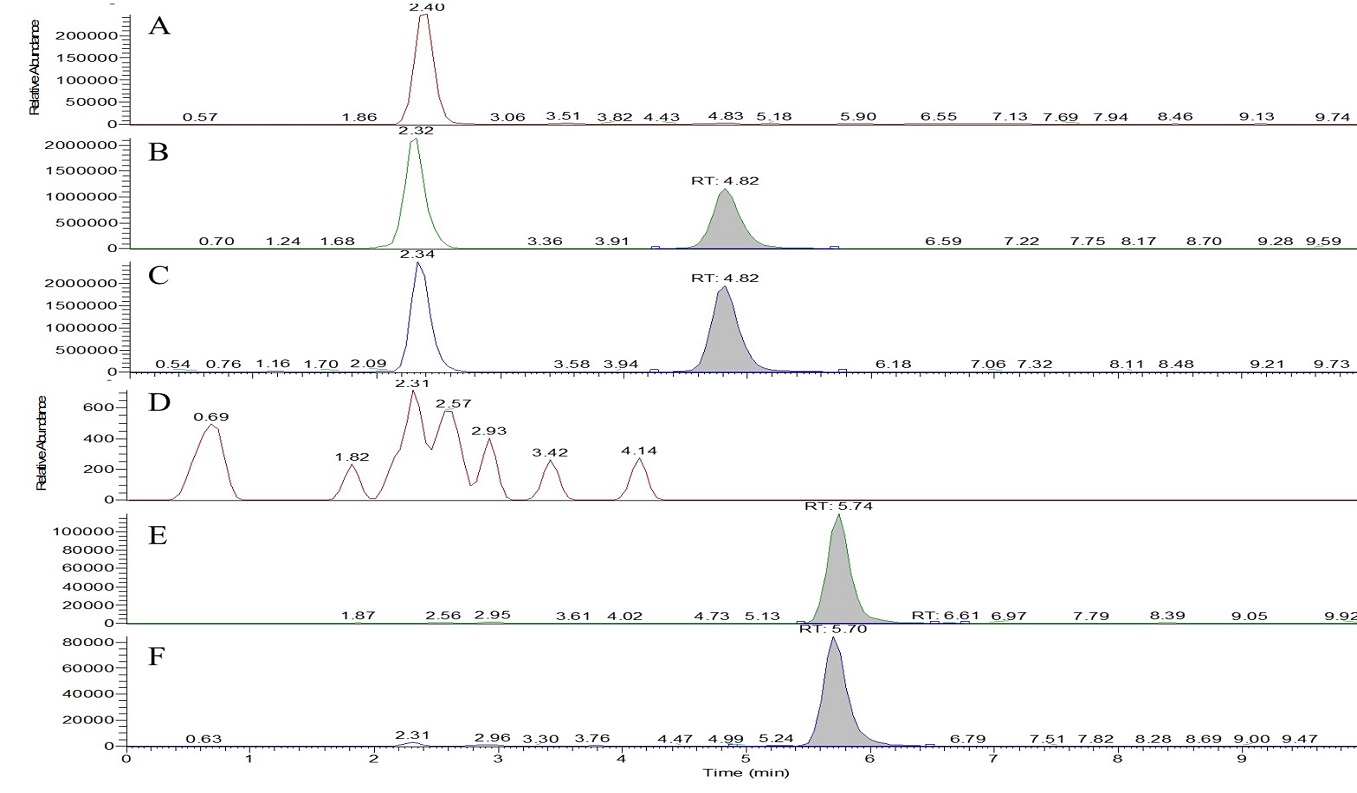
**

**Figure S4. Typical SIM chromatograms for the determination of COS 2 and COS 3 in kidney: blank matrix (A) (D); blank matrix spiked with COS 2 (B) and COS 3 (E); after intragastric administration of 500 mg/kg COS 2 (C) and after intragastric administration of 500 mg/kg COS 3 (F).**

**
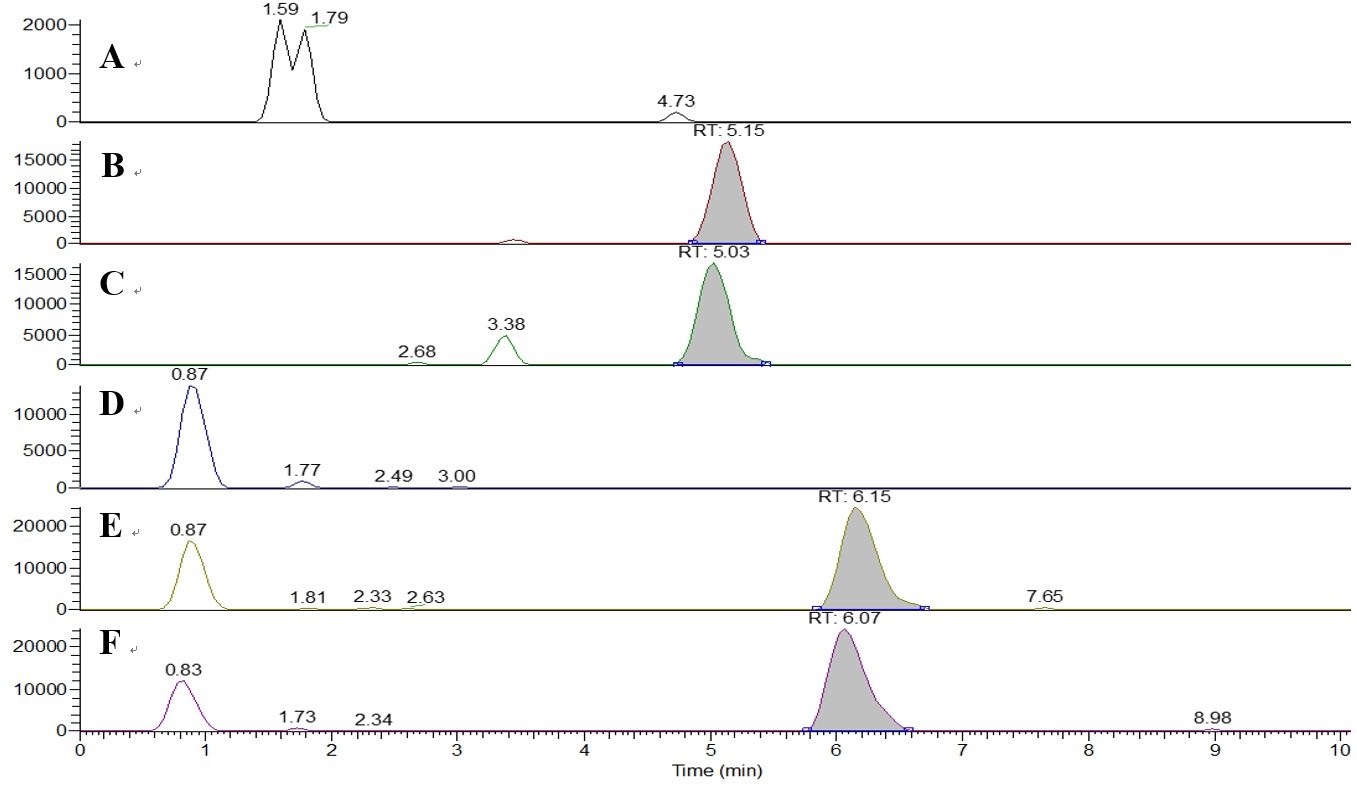
**

**Figure S5. Typical SIM chromatograms for the determination of COS 2 and COS 3 in lung: blank matrix (A) (D); blank matrix spiked with COS 2 (B) and COS 3 (E); after intragastric administration of 500 mg/kg COS 2 (C) and after intragastric administration of 500 mg/kg COS 3 (F).**

**
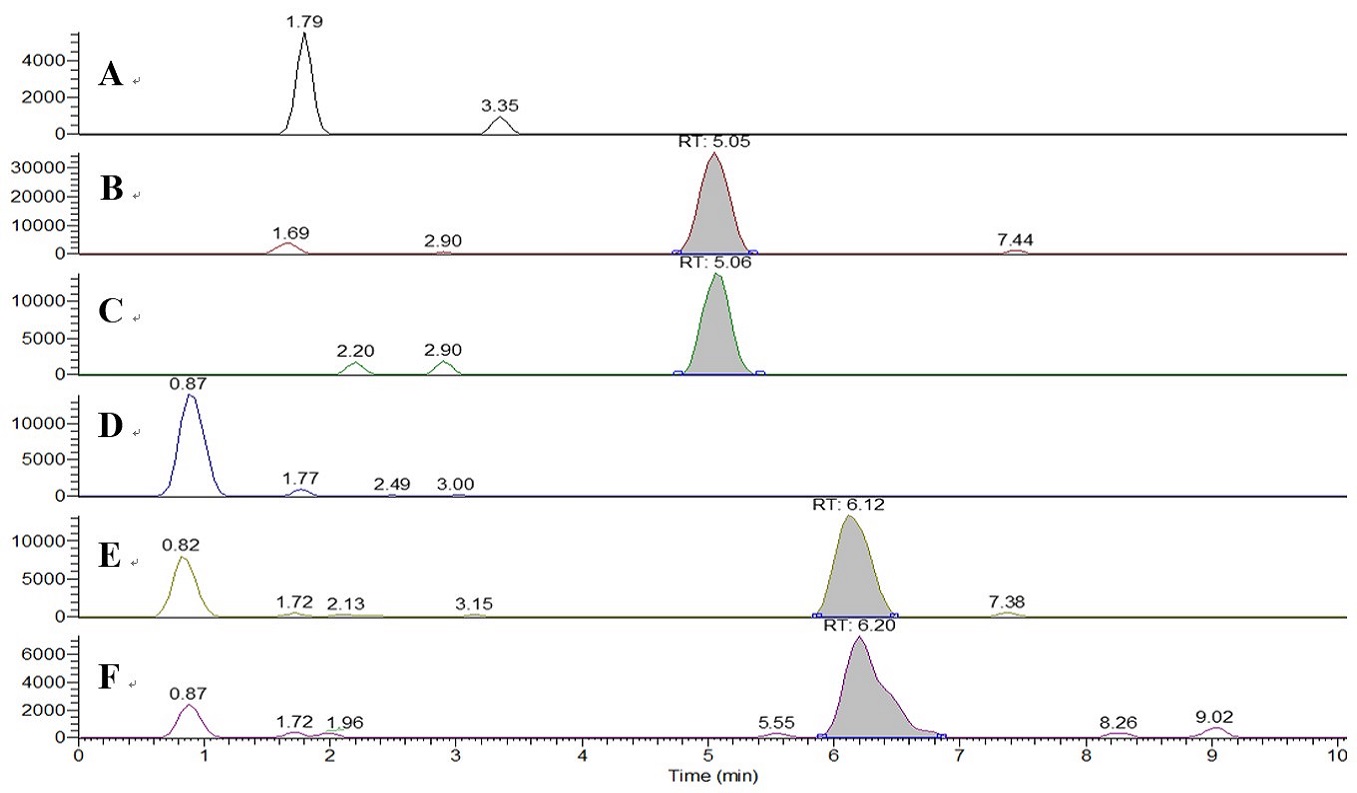
**

**Figure S6. Typical SIM chromatograms for the determination of COS 2 and COS 3 in spleen: blank matrix (A) (D); blank matrix spiked with COS 2 (B) and COS 3 (E); after intragastric administration of 500 mg/kg COS 2 (C) and after intragastric administration of 500 mg/kg COS 3 (F).**

**
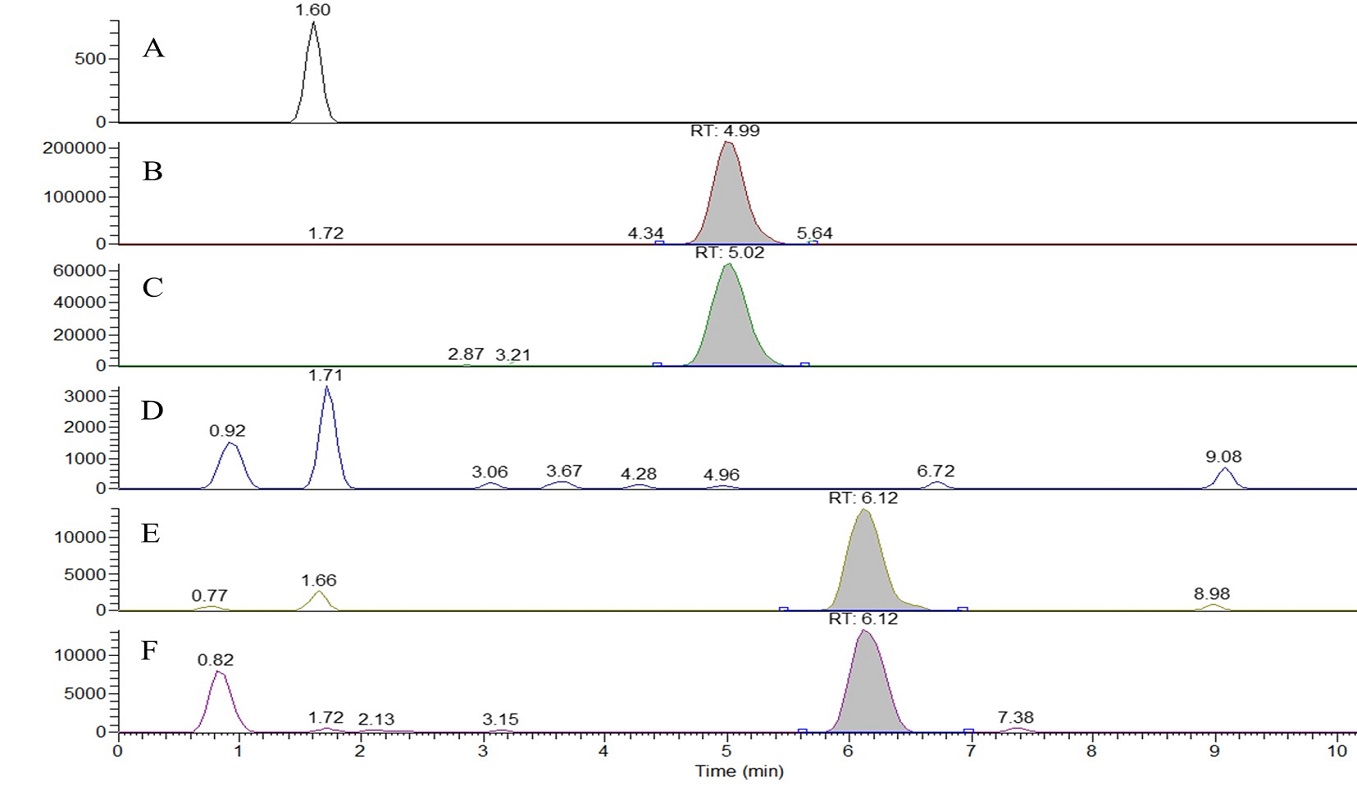
**

**Figure S7. Typical SIM chromatograms for the determination of COS 2 and COS 3 in pancreas: blank matrix (A) (D); blank matrix spiked with COS 2 (B) and COS 3 (E); after intragastric administration of 500 mg/kg COS 2 (C) and after intragastric administration of 500 mg/kg COS 3 (F).**

**
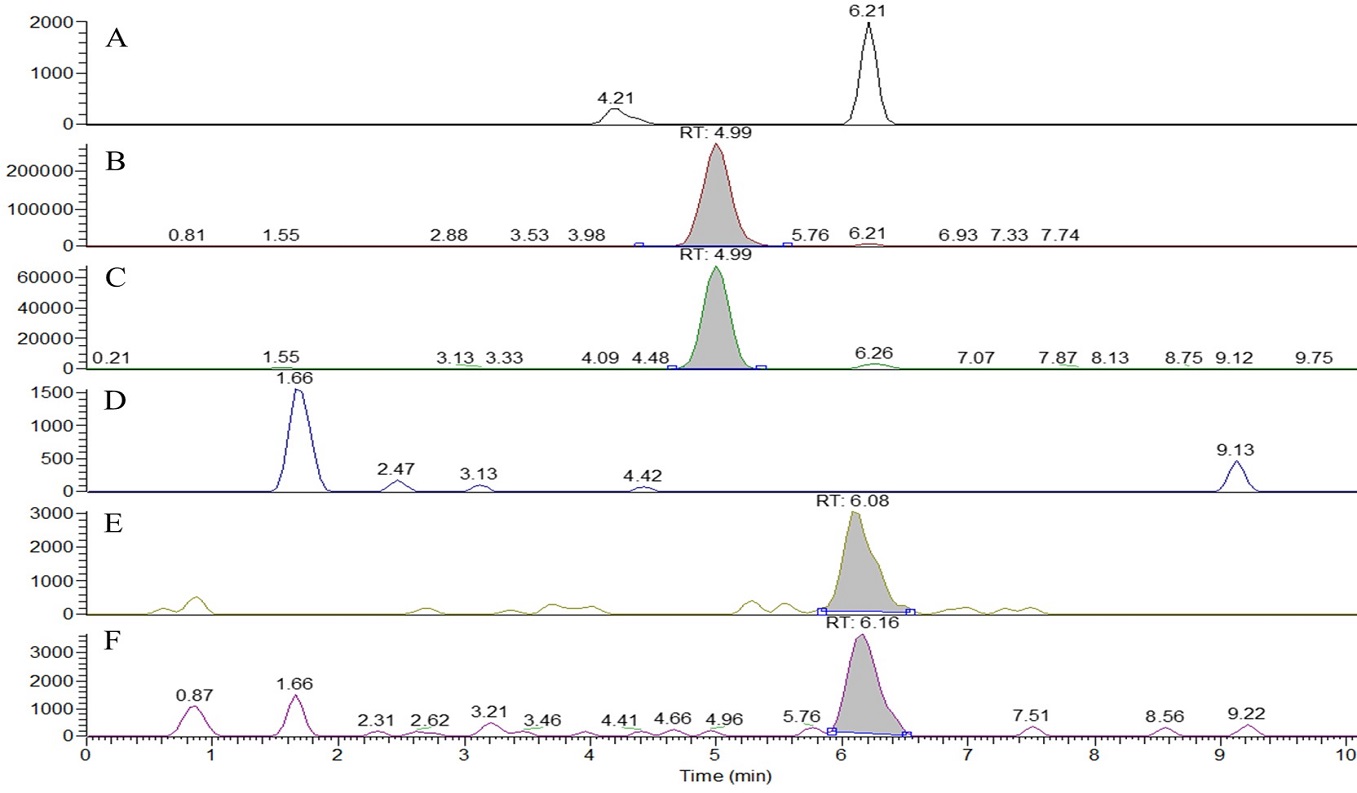
**

**Figure S8. Typical SIM chromatograms for the determination of COS 2 and COS 3 in cerebellum: blank matrix (A) (D); blank matrix spiked with COS 2 (B) and COS 3 (E); after intragastric administration of 500 mg/kg COS 2 (C) and after intragastric administration of 500 mg/kg COS 3 (F).**

**
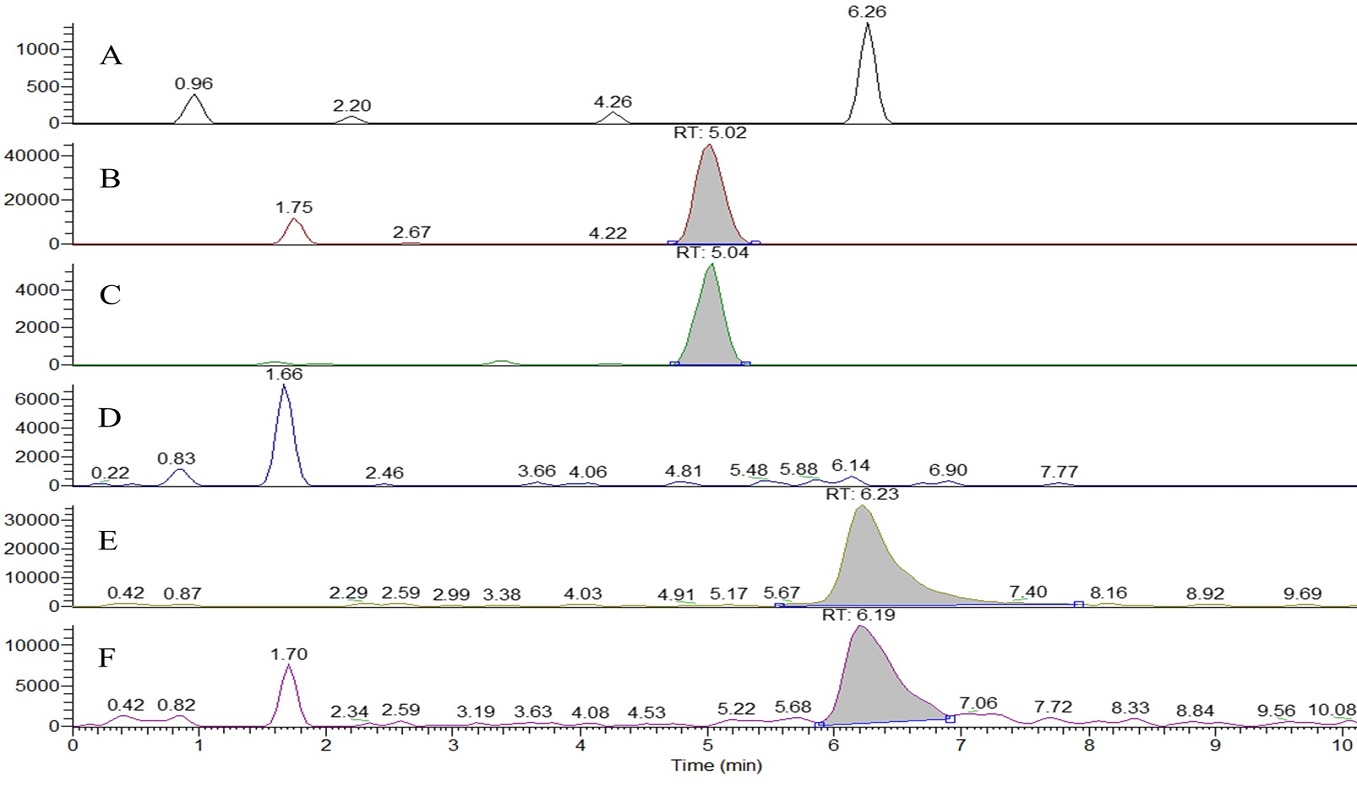
**

**Figure S9. Typical SIM chromatograms for the determination of COS 2 and COS 3 in brain: blank matrix (A) (D); blank matrix spiked with COS 2 (B) and COS 3 (E); after intragastric administration of 500 mg/kg COS 2 (C) and after intragastric administration of 500 mg/kg COS 3 (F).**


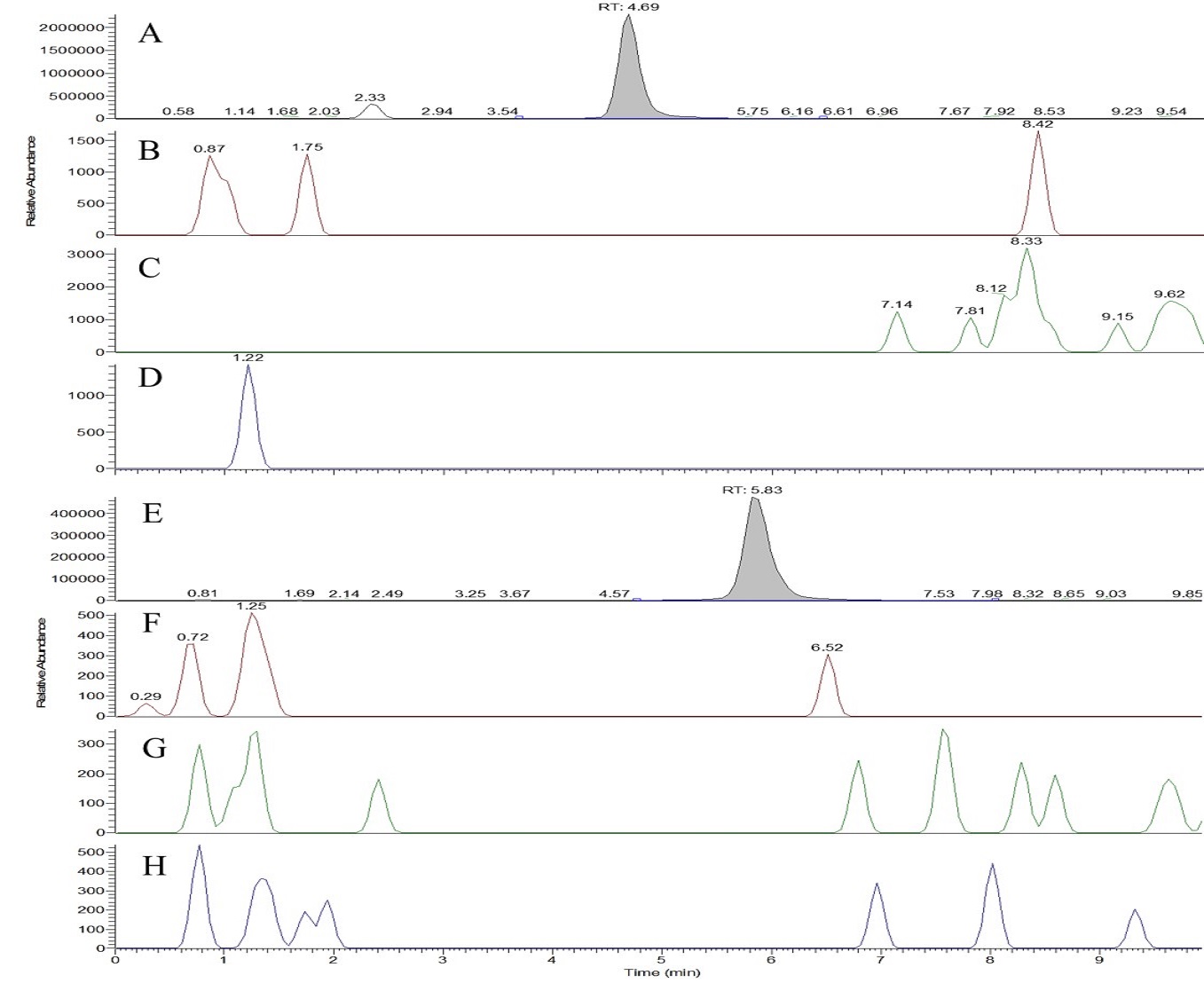


**Figure S10. SIM chromatograms for the determination of COS 2 and COS 3 in serum: high QC samples (A) (E); blank serum samples (C-E) (F-H).**
